# Integrating Genome-Wide Association Study with RNA-Sequencing Reveals *HDAC*9 as a Candidate Gene Influencing Loin Muscle Area in Beijing Black Pigs

**DOI:** 10.3390/biology11111635

**Published:** 2022-11-08

**Authors:** Renda Hou, Li Chen, Xiance Liu, Hai Liu, Guohua Shi, Xinhua Hou, Run Zhang, Man Yang, Naiqi Niu, Lixian Wang, Longchao Zhang

**Affiliations:** 1 Institute of Animal Science, Chinese Academy of Agricultural Sciences, Beijing 100193, China; 2 Chongqing Academy of Animal Science, Chongqing 402460, China; 3 Beijing Heiliu Animal Husbandry Technology Co., Ltd., Beijing 102211, China

**Keywords:** Beijing Black pig, *HDAC*9, genome-wide association study, loin muscle area, transcriptome

## Abstract

**Simple Summary:**

In this study, the genomic variants associated with LMA in Beijing Black pigs revealed a total of 329 differentially expressed genes relating to LMA. Integrating a GWAS with RNA sequencing revealed *HDAC*9 as a crucial candidate gene for this trait. KEGG analysis using RNA-seq data indicated that the DEGs were mainly enriched in the JAK/STAT and oxytocin signaling pathways. We believe that our study makes a significant contribution to the literature because, although many candidate genes related to LMA have been reported, the molecular markers and candidate genes for LMA in Beijing Black pigs have not been identified.

**Abstract:**

Loin muscle area (LMA) is an important meat production trait and plays a key role in determining carcass leanness. Genome-wide association study (GWAS) and RNA sequencing (RNA-seq) analysis were used to identify candidate LMA genes in Beijing Black pigs, a popular breed among consumers in northern China. Ten single nucleotide polymorphisms (SNPs) in *sus scrofa chromosome* (SSC) 9 were significantly associated with LMA. These SNPs were mapped to a 2.90 Mb (84.94–87.84 Mb) region. A total of 11 annotated genes were mapped on this region, namely *MEOX*2, *CRPPA*, *SOSTDC*1, *LRRC*72, *ANKMY*2, *BZW*2, *TSPAN*13, *AGR*2, *AHR*, *SNX*13, and *HDAC*9. In addition, RNA-seq analysis was performed between the high- and low-LMA groups, and 329 differentially expressed genes (DEGs) were identified. Further, Kyoto Encyclopedia of Genes and Genomes analysis based on DEGs revealed that the JAK/STAT signaling pathway and oxytocin signaling pathway may be responsible for LMA. Both GWAS and RNA-seq analysis identified the *HDAC*9 gene, indicating that it may be an important candidate gene affecting LMA in Beijing Black pigs. The findings provide valuable molecular insights into the mechanisms that influence LMA content in pigs, which can be utilized in targeted approaches to enhance meat quality and commercial profitability.

## 1. Introduction

The loin muscle is one of the most economically valuable parts of pork. The loin muscle area (LMA) is an important factor in estimating lean meat percentage [1], which is a key indicator in the evaluation of carcass quality grading [2]. It is widely believed that genetics play the largest role in the development of the loin muscle. The heritability of LMA ranges from 0.35 to 0.47 [3,4], indicating that this trait can be improved by genetic methods. Given the importance of LMA in production, there have been several molecular studies on LMA traits in recent years, and a large number of candidate genes and single nucleotide polymorphisms (SNPs) have been identified. To date, 415 quantitative trait loci (QTLs) associated with LMA have been reported in the pig QTL database (http://www.animalgenome.org/cgi-bin/QTLdb/SS/index, accessed on 24 August 2022). Previous studies have identified a few molecular markers and candidate genes for pork LMA (e.g., *FGF*21 [5], *FUBP*3 [6], and *DRGA0016148* [7]) using genome-wide association studies (GWAS). The above markers and candidate genes have played important roles in the genetic improvement of LMA.

Developed in the 1960s from Berkshire, Large White, and Chinese indigenous pig breeds (Dingxian, Shenxian, Zhouxian, respectively) [8], the Beijing Black pig has become an important pig breed in northern China due to the good flavor of its meat. Although many candidate genes related to loin muscle area have been reported, the molecular markers and candidate genes for LMA in Beijing Black pigs have yet to be identified. Therefore, the aim of this study is to identify putative genetic loci or candidate genes for LMA in Beijing Black pigs using GWAS and RNA sequencing (RNA-seq) joint analysis.

## 2. Materials and Methods

### 2.1. Animals and LMA Measurement

In this study, a total of 650 Beijing Black pigs were raised by Beijing Hei6 Husbandry Technology Co., Ltd., Jintun, China, and slaughtered at 210 ± 40 days of age. The loin muscle area at the last rib was recorded using sulfuric acid paper and scanned using a Lenovo M700 scanner at 300 pixels. The actual loin muscle area was subsequently calculated by dividing the pixel value obtained by selecting the loin muscle area contour with PS2020 by the standard pixel value.

### 2.2. Genotyping and Quality Control

Using the salting-out procedure, DNA for genomic analysis was isolated from all samples [9]. All the animals were genotyped using the Illumina porcine 50 K BeadChip (Illumina. San Diego, CA, USA) containing 50,697 SNPs. In compliance with the quality control requirements, SNPs and samples with call-rates lower than 90 percent or SNPs with a minor allele frequency (MAF) lower than 0.05 across all samples genotyped with each chip were excluded. PLINK version 1.90 was used to perform these quality controls.

### 2.3. Genome-Wide Association Study

In order to conduct a GWAS, a single-locus, mixed linear model GWAS was employed (EMMAX: Efficient Mixed-Model Association eXpedited). It has been demonstrated that the EMMAX statistic test approach performs better than the principal component analysis and genomic control when sample structure (stratification and relatedness) is taken into account [10]. In our analysis, the sex and slaughter batch were used as fixed effects, and the slaughter age was used as a covariate. The genotype data were used in EMMAX to construct the n × n genetic matrix of identity between individuals. The model used can be expressed as

(1) y=Xβ+Zμ+e

where *y* is n × 1 the vector of observed phenotypic values of the animals, *X* is n × f matrix of fixed effects, *β* is q × 1 vector representing coefficients of the fixed effects, *Z* is n × t matrix relating the instances of the random effect to LMA, *μ* is random animal effects, and *e* is the residual effect.

### 2.4. Total RNA Isolation, Purification, and Quantification

The high (H) and low (L) LMA groups were made up of eight animals with a high LMA (42.04 cm^2^) and eight animals with a low LMA (24.20 cm^2^) (Table S1), and the mean age at slaughter for the H- and L-LMA groups was 210 ± 10 days. Following the manufacturer’s instructions, the TRIzol reagent (Invitrogen, Carlsbad, CA, USA) was used to extract total RNA from Beijing Black pig longissimus dorsi muscles. An ND-1000 spectrophotometer (NanoDrop, Wilmington, DE, USA) was used to measure RNA concentration and quality. RNA integrity was assessed using an Agilent 2100 with RIN number >7.0.

### 2.5. cDNA Library Construction and Sequencing

Total RNA was purified using two rounds of purification. The RNA was purified using poly T oligo-attached magnetic beads, lysed into small divalent fragments under high-temperature conditions, reverse-transcribed to cDNA, and then used to synthesize U-labeled double stranded DNA with *Escherichia coli* DNA polymerase I, Rnase H, and dUTP. The A-base was added to the blunt ends of each strand and then ligated to Illumina multiplex barcode adapters, which included custom unique molecular identifiers for minimizing sequence-dependent bias and amplification noise [11]. U-labeled secondary strands were treated with heat-labeled UDG enzymes and amplified using polymerase chain reaction (PCR). The average insert size of the final cDNA library was 300 bp (±50 bp). Two-terminal sequencing was performed on an Illumina HiSeq 4000 (LC Bio, Hangzhou, China), according to the protocol recommended by the supplier.

### 2.6. Data Quality Control and Differential Gene Expression Data Analysis

The Illumina sequencing platform was used for this project. Prior to assembly, reads that were determined to be of poor quality (reads that included sequencing adaptors, sequencing primers, or a quality score of <20) were eliminated. The filtered reads were mapped to the porcine genome using HISAT2 v2.1.0 [12] (*Sus scrofa* 11.1). In order to convert the aligned file to BAM format, SAMtools v1.6.0 [13] was used. The raw counts of genes and transcript levels were calculated using StringTie [14]. DESeq2 was used for differential expression analysis [15]. Using the Benjamini–Hochberg method [16] with adjusted *p* < 0.05, genes with an expression fold change (FC) > 2 were considered to be differentially expressed genes (DEGs). Principal component analysis (PCA) and heatmaps were performed on the normalized gene expression data to visualize the expression intensity values of the resulting muscle area transcripts.

### 2.7. Gene Ontology (GO) Annotation and Kyoto Encyclopedia of Genes and Genomes (KEGG) Pathway Analysis of DEGs

All DEGs were mapped to GO terms in the GO database [17] (http://www.geneontology.org, accessed on 22 July 2022). Based on the hypergeometric test, GO terms in DEGs that were significantly enriched were found, with *p* < 0.05 set as the norm. Only DEGs with a *p* < 0.05 in DESeq2 were used for further analysis in this study. Pathways in the KEGG database were found using the Kobas functional annotation tool (v3.0).

### 2.8. Quantitative Real-Time PCR (qRT-PCR) Validation

Quantitative real-time PCR was performed on 10 randomly chosen genes to ensure that the sequencing findings were accurate. Total RNA was reverse-transcribed according to the manufacturer’s instructions using a PrimeScript RT kit (TaKaRa, Kusatsu, Japan). The qRT-PCR was used to normalize the transcript levels of the examined genes to those of *GAPDH* and to compute the 2^−△△ct^ values [18,19]. QuantStudio 7 Flex (ABI, USA) was used to perform qRT-PCR; the reactions were conducted in triplicate. Amplification conditions included 40 cycles of 95 °C for 5 s, 60 °C for 34 s, 95 °C for 15 s, 60 °C for 1 s, and 95 °C for 15 s. Three replications of each experiment were carried out. Primer 5.0 was used to create primers based on sequence data from the NCBI database (Table S2).

## 3. Results

### 3.1. Genome-Wide Association SNPs for LMA

**Figure 1:**
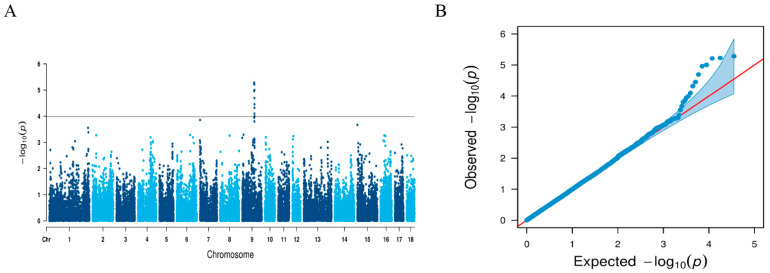
GWAS for LMA. (**A**) Manhattan plots for LMA. The red horizontal line indicates the FDR threshold (1.04 × 10^−4^). (**B**) Quantile–quantile plot of SNPs after quality control in genome-wide association analysis for LMA.

The mean and standard deviations of LMA of the 650-strong Beijing black pig population were 31.5 cm^2^ and 4.69 cm^2^, respectively. A total of 35,401 SNPs were in the final dataset used for the GWAS after quality control (Table S3). For GWAS, a threshold of 1.04 × 10^−4^ (3.69/35,401) was used. Figure 1 shows the Manhattan plot and the quantile–quantile (Q–Q) plot from this GWAS. When performing a GWAS, dividing the population into groups may yield false-positive results. The Q–Q plot demonstrates that there was no clear systematic bias in the traits as a whole. From the GWAS on SSC9, 10 genome-wide important SNPs that affect LMA were identified (Table 1).

**Figure 2:**
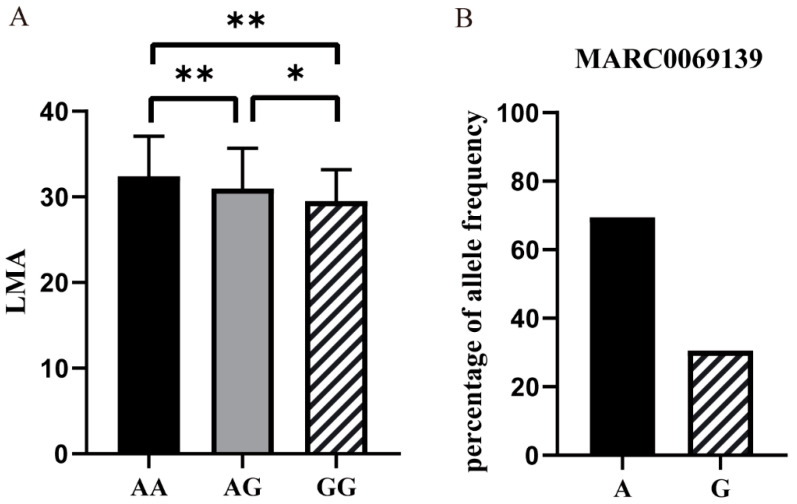
Identification of MARC0069139 using GWAS of loin muscle area. (**A**) The difference analysis among three genotypes of MARC0069139 in Beijing Black pigs. * *p* < 0.05, ** *p* < 0.01. (**B**) Allele frequencies of MARC0069139 in Beijing Black pigs.

On SSC9, 10 SNPs displayed significant genome-wide associations with LMA. These SNPs were mapped to a 2.90 Mb (84.94–87.84 Mb) region. The strongest association of these SNPs was with MARC0069139 at 87.84 Mb. A total of 11 annotated genes were mapped on this region. They were mesenchyme homeobox 2 (*MEOX*2), CDP-L-ribitol pyrophosphorylase A (*CRPPA*), sclerostin domain containing 1 (*SOSTDC*1), leucine rich repeat containing 72 (*LRRC*72), ankyrin repeat and MYND domain containing 2 (*ANKMY*2), basic leucine zipper and W2 domains 2 (*BZW*2), tetraspanin 13 (*TSPAN*13), anterior gradient 2 (*AGR*2), aryl hydrocarbon receptor (*AHR*), sorting nexin 13 (*SNX*13), and histone deacetylase 9 (*HDAC*9). For MARC0069139, individuals with the AA genotype had a higher LMA than those with the GG genotype (*p* < 0.01, Figure 2A). The frequency of the A allele was higher than that of the G allele (Figure 2B).

### 3.2. RNA-Seq Data

Sixteen cDNA libraries from the H and L groups were sequenced, yielding 712,114,178 clean reads. An average of 44,507,136 reads were obtained for each sample (ranging from 37,346,508 to 52,970,650 reads). The quality values of Q20 and Q30 were 99.97% and 98.10 %, respectively. Total RNA-seq resulted in an average of 97.01% of reads that passed the quality control filters. An average of 97.03% of the reads were mapped to the reference genome (*Sus scrofa* 11.1). These results indicated that the sequencing data in this study were reliable and could be used for subsequent bioinformatics analysis (Table 2).

### 3.3. DEGs between Higher and Lower LMA Groups

**Figure 3:**
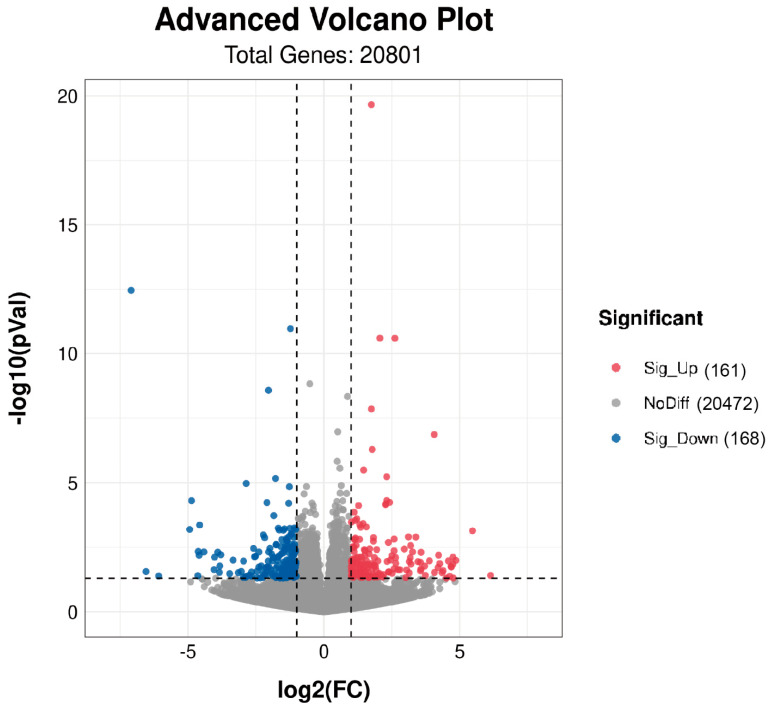
Volcano Plot of DEGs in longissimus dorsi muscle comparing H (high) group and L (low) group. The x-axis shows the values of log2 (fold change), while the average expression values of log10 (*p*-Value) are displayed by the y-axis. The red and blue dots represent the significantly differentially expressed transcripts (*p*-Value < 0.05) comparing the L group, with red for upregulated genes and blue for downregulated genes. The gray dots indicate the transcripts with expression levels which are not statistically significant (*p*-Value > 0.05).

**Figure 4:**
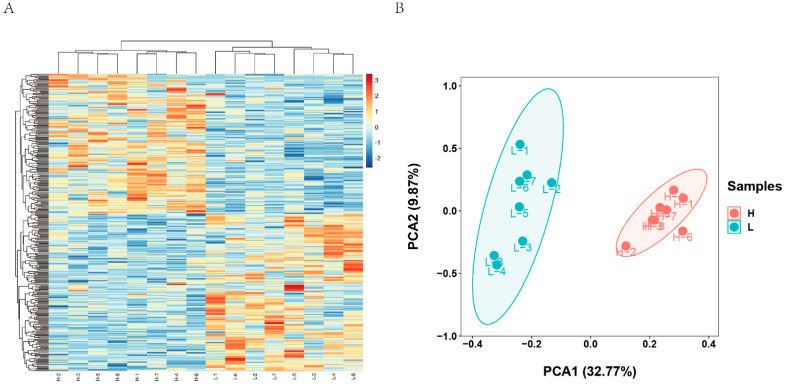
Expression profiles analysis of DEGs. (**A**) Heat map of DEGs between H (high) and L (low) groups. (**B**) PCA of DEGs between H (high) and L (low) groups.

Gene expression was detected using counts. We identified 329 DEGs associated with LMA in group H and L (*p* < 0.05, fold change ≥ 2). Compared to the L group, 161 DEGs were upregulated and 168 DEGs were downregulated in group H (Figure 3, Table S4). Heat maps (Figure 4A) were generated and PCA (Figure 4B) was performed by normalizing the gene expression data. The samples in groups H and L were clearly clustered into two branches of high and low groups, and genes with similar expression patterns were clustered together, indicating significant intersample differences and good reproducibility.

### 3.4. GO Enrichment and KEGG Pathway Analysis

**Figure 5:**
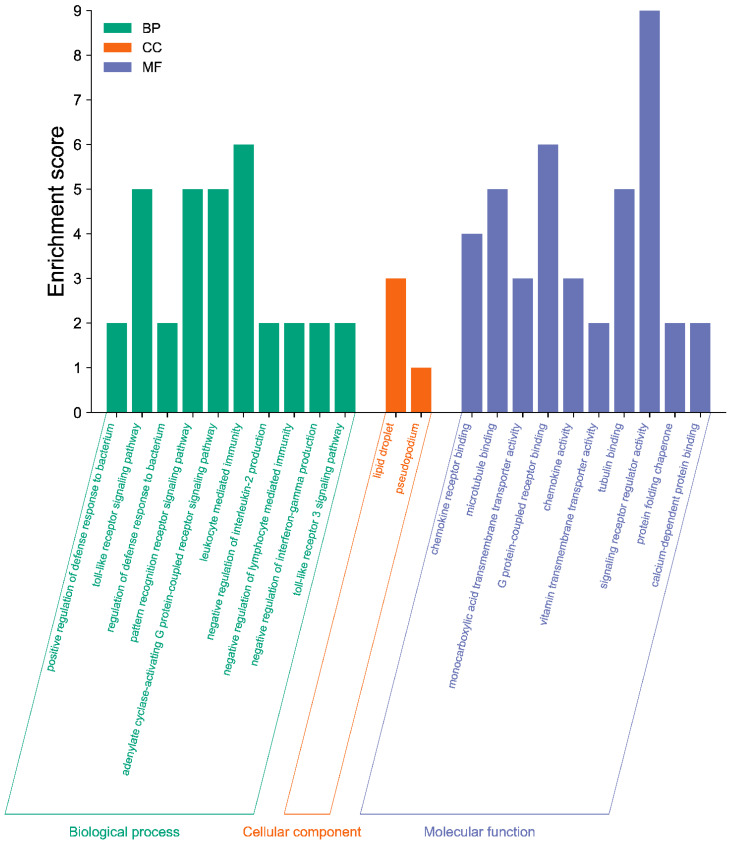
Most highly enriched GO terms of DEGs between H group and L group. The y-axis displays the number of DEGs, and the x-axis represents the GO terms. The bar colors correspond to different GO categories, with purple for molecular function, green for biological process, and orange for cellular component.

To determine the functions of the DEGs, GO and KEGG enrichment analyses were carried out. The results of the GO analysis showed that the DEGs were mainly involved in the regulation of the defense response to bacteria, toll-like receptor signaling pathway, leukocyte mediated immunity, cell adhesion mediator activity, and chemokine receptor binding (Figure 5). In addition, some DEGs enriched in GO terms were correlated with myogenesis, such as negative regulation of myoblast fusion, muscle atrophy, and leptin receptor activity (Table S5).

**Figure 6:**
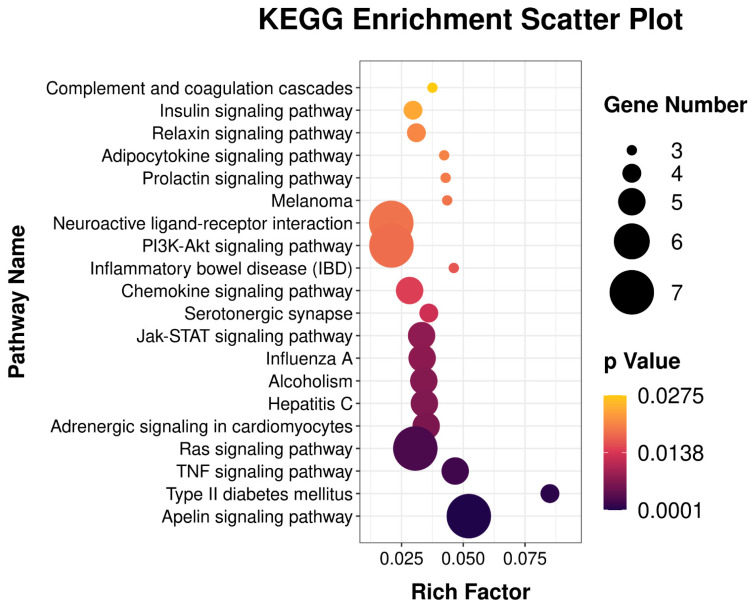
The column diagrams for Kyoto Encyclopedia of Genes and Genomes (KEGG) analysis of DEGs. The x-axis represents the numbers of DEGs. The y-axis represents the functions of pathways. The color depth represents *p*-value.

The KEGG tool was used to systematically analyze cellular metabolic pathways and gene product functions to study the complex functions of genes. The 20 most enriched pathways (*p* < 0.05) are shown in Figure 6. KEGG analysis showed that the DEGs were enriched in pathways related to myogenesis, including glycolipid metabolism, and the JAK/STAT, PI3K-Akt, adipocytokine, Rap1, and oxytocin signaling pathways (Table S6).

### 3.5. Validation of Gene Expression Using qRT-PCR

**Figure 7:**
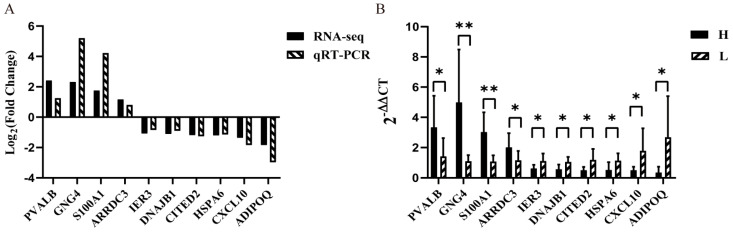
Comparison of gene expression. (**A**) Comparison of the fold change between RNA-seq and qRT-PCR. (**B**) Gene expression of H and L group by qPCR. * *p* < 0.05, ** *p* < 0.01.

Ten DEGs (*PVALB*, *GNG*4, *S100A*1, *ARRDC*3, *IER*3, *DNAJB*1, *CITED*2, *HSPA*6, *CXCL*10, and *ADIPOQ*) were used to determine the difference in gene expression between the H and L groups using qRT-PCR to validate the accuracy of transcriptome sequencing (Figure 7B). The qRT-PCR and RNA-seq data revealed a consistent pattern of gene expression (Figure 7A).

## 4. Discussion

There have been several reports of quantitative trait loci (QTL) for LMA. For instance, Edwards et al. [20] used 510 Duroc × Pietran F2 animals genotyped for 124 microsatellite markers that were uniformly spaced across the genome and detected a significant QTL that affects LMA and the related backfat trait. Furthermore, on SSC12 the SRY-related HMG-box 15 (*SOX*15) locus was displayed as a QTL for LMA in GWAS using the Landrace × KNP F2 intercross populations [21]. Using the Meishan × Yorkshire F2 population, QTLs for LMA were mapped on SSC 1, 2, 4, 11, 12, and 14 [22]. Ponsuksili et al. identified an LMA-related QTL (SSC12, 90–97 cM) in the Duroc × Berlin Miniature Pig F2 population [23]. In the current study, QTL signals of LMA were mapped using GWAS on SSC9 of Beijing Black pigs. These results suggest that LMA-regulated genes exhibit significant population specificity.

On SSC9, previous studies have mapped QTLs for LMA in the 45.3–109.6 Mb region. Our study identified 10 genome-wide significant SNPs across 84.94–87.84 Mb, with regions of overlap with those reported in previous reports. This region contains 11 annotated genes. *MEOX*2 is a key gene in the regulatory framework controlling limb muscle development in vertebrate embryos [24]. The loss of *MEOX*2 results in smaller limb muscles that harbor reduced numbers of myofibers [25]. Mutations in the *CRPPA* gene can produce a deficit of functioning α-dystroglycan and damaged muscle fibers, which impairs skeletal muscle development, structure, and function [26]. In addition to generating dystroglycanopathies, *CRPPA* mutations have also been linked to limb girdle muscular dystrophy, Walker–Warburg syndrome, and muscle–eye–brain disease [27,28]. When *SOSTDC*1 is knocked down, it promotes the migration of linear morphea fibroblasts [29]. A previous study suggested that *ANKMY*2 plays a key role in the regulation of Shh signaling in vivo [30]. *BZW*2 has been shown to promote fibrosarcoma tumor growth [31]. Downregulation of *BZW*2 results in apoptosis and a reduction in cell cycle progression and proliferation [32]. *TSPAN*13 reduces proliferation and invasion and enhances apoptosis of breast cancer cells in vitro and in vivo [33]. *AGR*2 is a promoter of cancer cell proliferation, invasion and survival, chemotherapy resistance, metastasis, and tumor growth [34,35,36,37,38]. *AHR* enables cells to adapt to changing conditions by sensing compounds from the environment, diet, microbiome, and cellular metabolism, which plays an important role in development of and immunity to cancer [39,40,41,42]. *SNX*13 has been shown to be a good candidate for milk production traits in cattle [43]. *HDAC*9 is highly expressed in skeletal muscle [44].

In the current study, we identified a total of 329 DEGs relating to LMA. Several DEGs, such as adiponectin, C1Q and collagen domain containing (*ADIPOQ*), Interferon Alpha Inducible Protein 6 (*IFI*6), and leptin receptor (*LEPR*), were previously reported to affect muscle fiber growth and development in animals. One of the biologically active adipokines, *ADIPOQ*, is essential for mediating antidiabetic and anti-atherogenic actions [45]. *ADIPOQ* may also play a significant role in the regulation of muscle fiber type, as evidenced by the fact that it is expressed by skeletal muscle fibers and affects the types of muscle fiber [46]. When compared to other tissues, muscle and bone have higher levels of *IFI*6 expression, suggesting that this gene is most active in these tissues. Kayan et al. demonstrated that *IFI*6 was significantly associated with LMA using the F2 population of Duroc × Pietran pigs [47]. In previous studies, *LEPR* has been used as a candidate gene for LMA [48] and plays an important role in muscle atrophy [49]. According to the DEGs, the JAK/STAT and oxytocin signaling pathways (known to be associated with myofiber growth and development) were enriched. Studies have shown that the JAK/STAT pathway controls the myogenic development of adult satellite cells (MuSCs), a cell type that is essential for postnatal skeletal muscle growth and damage repair [50]. Inhibiting oxytocin signaling in young animals inhibits muscle regeneration, whereas systemic administration of oxytocin promotes muscle regeneration swiftly by increasing aged muscle stem cell activation/proliferation via the MAPK/ERK signaling pathway [51].

Of the 329 DEGs, *HDAC*9 was shown to be a candidate gene for LMA in both GWAS and RNA-seq analyses. To date, 18 histone deacetylases have been identified in mammals and are divided into four classes. Among them, Class II HDACs (*HDAC*4, *HDAC*5, *HDAC*7, and *HDAC*9) are highly expressed in skeletal muscle and directly bind *MEF*2, repressing expression of MEF2-dependent genes [44]. The ability of MRFs to transform fibroblasts into myoblasts is one of their most notable and distinguishing features. When *MEF*2 is cotransfected with MRFs, this ability is considerably improved [52]. Previous studies have shown that in vitro and in vivo the *HDAC*9 gene is a direct transcriptional target of *MEF*2. *HDAC*9 proteins can bind to *MEF*2 proteins and inhibit their transcriptional activity. The transcriptional repressor, *HDAC*9, thereby generates a negative feedback loop in the muscle development transcriptional circuitry [53]. The transcriptome results indicated a lower expression of *HDAC*9 in the high LMA group compared to the low LMA group, consistent with findings of previous reports. *HDAC*9 must therefore play an important role in LMA in Beijing Black pigs.

## 5. Conclusions

This study describes the genomic variants associated with LMA in Beijing Black pigs. Our results suggest that *MEOX*2, *CRPPA*, *SOSTDC*1, *LRRC*72, *ANKMY*2, *BZW*2, *TSPAN*13, *AGR*2, *AHR*, *SNX*13, and *HDAC*9 on SSC9 may play important roles in affecting LMA. Furthermore, KEGG analysis using RNA-seq data indicated that the DEGs were mainly enriched in the JAK/STAT and oxytocin signaling pathways. Integrating GWAS with RNA-seq revealed that *HDAC*9 is a crucial candidate gene for this trait. The identified variations provide valuable molecular information that may be harnessed to increase LMA in pork. This study provides new insights into the molecular mechanisms that regulate LMA in pigs.

## Figures and Tables

**Table 1:** Description of SNPs significantly associated with LMA on SSC9.

Marker	Position ^a^	Rs ^b^	*p*-Value	Var (%) ^c^
WU_10.2_9_93503815	84,937,219	rs336943465	5.19 × 10^−6^	2.08
ALGA0054101	85,233,732	rs81413966	1.10 × 10^−5^	1.94
ASGA0043938	85,259,596	rs81413967	6.12 × 10^−6^	2.05
ASGA0043959	86,791,976	rs81414045	7.94 × 10^−5^	1.56
MARC0073290	86,843,385	rs81259452	5.91 × 10^−6^	2.06
MARC0046912	86,880,793	rs81238869	2.02 × 10^−5^	1.82
MARC0065750	86,920,421	rs81253220	9.91 × 10^−6^	1.96
MARC0055652	87,156,018	rs81245907	3.52 × 10^−5^	1.71
ASGA0043969	87,327,648	rs81414101	4.76 × 10^−5^	1.66
MARC0069139	87,837,573	rs81256749	1.01 × 10^−4^	1.51

^a^ Data from Sus scrofa Build 11.1; ^b^ Rs, reference SNP; ^c^ Var (%), phenotypic variation explained by the SNP.

**Table 2:** RNA sequencing data analysis.

Sample_Name	Raw_Reads	Clean_Reads	Q30 (%)	GC_Content (%)	Mapped (%)
H1	42,921,002	41,411,838	98.03	52.00	96.48
H2	51,521,550	50,088,198	98.55	51.50	97.22
H3	49,671,858	47,485,598	97.96	52.00	95.60
H4	45,497,868	44,432,658	97.49	52.00	97.66
H5	40,988,148	39,848,532	97.98	52.00	97.22
H6	39,841,846	38,699,270	97.97	52.00	97.13
H7	47,118,756	45,666,934	98.21	51.00	96.92
H8	50,646,242	49,107,270	97.97	52.00	96.96
L1	54,959,674	52,970,650	98.22	52.00	96.38
L2	52,534,364	51,224,866	98.06	52.00	97.51
L3	45,241,998	43,696,868	98.32	51.00	96.58
L4	42,172,534	41,077,200	98.25	51.50	97.40
L5	41,125,930	39,957,600	98.17	52.00	97.16
L6	38,604,910	37,465,804	98.13	52.00	97.05
L7	52,999,242	51,634,384	98.00	53.00	97.42
L8	38,195,034	37,346,508	98.25	52.00	97.78

## Data Availability

The raw sequence data reported in this paper have been deposited in the Genome Sequence Archive (Genomics, Proteomics & Bioinformatics 2021) in the National Genomics Data Center (Nucleic Acids Res 2022), China National Center for Bioinformation/Beijing Institute of Genomics, Chinese Academy of Sciences (GSA: CRA008225) which is publicly accessible at https://ngdc.cncb.ac.cn/gsa.

## Supplementary Materials

The following supporting information can be downloaded at https://www.mdpi.com/article/10.3390/biology11111635/s1: Table S1. Descriptive statistics of loin muscle area from Beijing Black Pig population for RNA sequencing; Table S2. The details of primer design for qPCR of mRNA; Table S3. Distribution of SNPs after quality control and average distances on each chromosome; Table S4. Statistics of differentially expressed genes between H group and L group by RNA-seq; Table S5. Functional annotation clustering of differentially expressed genes in the longissimus dorsi muscle from Beijing Black pigs; Table S6. Pathway analysis for differentially expressed genes.
